# Heat Source Modeling in Selective Laser Melting

**DOI:** 10.3390/ma12132052

**Published:** 2019-06-26

**Authors:** Elham Mirkoohi, Daniel E. Seivers, Hamid Garmestani, Steven Y. Liang

**Affiliations:** 1 Woodruff School of Mechanical Engineering, Georgia Institute of Technology, Atlanta, GA 30332, USA; 2 Boeing Research and Technology, Ceramics, Extreme Environments & Metals, Huntsville, AL 35824, USA; 3 School of Materials Science and Engineering, Georgia Institute of Technology, Atlanta, GA 30332, USA

**Keywords:** temperature field, additive manufacturing, selective laser melting, heat source modeling

## Abstract

Selective laser melting (SLM) is an emerging additive manufacturing (AM) technology for metals. Intricate three-dimensional parts can be generated from the powder bed by selectively melting the desired location of the powders. The process is repeated for each layer until the part is built. The necessary heat is provided by a laser. Temperature magnitude and history during SLM directly determine the molten pool dimensions, thermal stress, residual stress, balling effect, and dimensional accuracy. Laser-matter interaction is a crucial physical phenomenon in the SLM process. In this paper, five different heat source models are introduced to predict the three-dimensional temperature field analytically. These models are known as steady state moving point heat source, transient moving point heat source, semi-elliptical moving heat source, double elliptical moving heat source, and uniform moving heat source. The analytical temperature model for all of the heat source models is solved using three-dimensional differential equations of heat conduction with different approaches. The steady state and transient moving heat source are solved using a separation of variables approach. However, the rest of the models are solved by employing Green’s functions. Due to the high temperature in the presence of the laser, the temperature gradient is usually high which has a substantial impact on thermal material properties. Consequently, the temperature field is predicted by considering the temperature sensitivity thermal material properties. Moreover, due to the repeated heating and cooling, the part usually undergoes several melting and solidification cycles, and this physical phenomenon is considered by modifying the heat capacity using latent heat of melting. Furthermore, the multi-layer aspect of the metal AM process is considered by incorporating the temperature history from the previous layer since the interaction of the layers have an impact on heat transfer mechanisms. The proposed temperature field models based on different heat source approaches are validated using experimental measurement of melt pool geometry from independent experimentations. A detailed explanation of the comparison of models is also provided. Moreover, the effect of process parameters on the balling effect is also discussed.

## 1. Introduction

Metal additive manufacturing (AM) processes are rapidly growing technologies that are utilized to build up 3D complex parts through a repetitive process of deposition of metallic powders and laser melting under the guidance of digital models [1,2]. Compared to traditional manufacturing such as machining, rolling, casting and etc., these rapid prototyping techniques offer single step, net shape processing of complex parts, reduction of production time due to the elimination of multi-step manufacturing and reduction in cost due to the reduction in material scrap rate [3,4].

The selective laser melting (SLM) process is the most common AM method for producing 3D complex, metallic structures [5]. The metallic micro-scale powders are liquified with a laser, forming a melt pool that penetrates the previously deposited build layers, joining the layers upon cooling [6]. SLM is growing in many fields such as medical industries to manufacture bones, implants, etc. [7]. Moreover, it is used in producing automobile and aerospace components to reduce the final part density and weight, in order to reduce energy usage [8,9].

Use of SLM parts in industries, however, requires time-consuming flaw detection including hig residual stress-induced crack initiation and growth [10,11], undesired microstructure-induced material properties [12], and high thermal stress induced delamination of the layers [13]. The main source of all abovementioned flaws in the SLM parts is a high temperature gradient induced by laser. Therefore, accuracy in the modeling of the temperature field is of great importance. To improve part quality, a thorough understanding of the real-time thermal characteristics is needed.

The complex multi-physics of the SLM process occurs at micro time and length scales. As a result of the complex phenomena involved in this process, relying on experimentation to understand the underlying physical aspects of the SLM process alone would be too time-consuming, costly, and complex [14,15,16]. Numerical models such as finite element models and finite volume models are used by many researchers, however, the simulation of the entire process could not be achieved in a traceable amount of time. Consequently, many simplifications in modeling should be undertaken [17,18,19,20]. In contrast, analytical models, validated by physical experiments, provide a means of both effectively understanding and optimizing the process by allowing for in-situ analysis as well as efficient optimization of process parameters [15,21,22].

Several efforts have been made to model the temperature field in the SLM process. These efforts can be classified into three main categories known as experimentation, numerical modeling, and analytical modeling. 

Experimental work has been done to measure the in-situ temperature using different setups. Thermocouples can be sited inside the substrate to measure the temperature [23,24]. Due to the existence of high temperature in metal AM, the thermocouple type R is recommended. It should be noted that the thermocouples can only measure the temperature inside the substrate rather than the build part. The thermal imager, infrared (IR) camera and pyrometer are the other available technologies that can be used in measuring the in situ temperature during the AM process [25,26]. These two approaches can also be used together to compensate for the disadvantages of each other. More information about different experimental setups can be found in different surveys [17,27].

Numerical models such as finite element model (FEM) and finite volume model (FVM) are used to simulate the AM process. Andreotta et al. employed a FEM to simulate the fluid flow and thermal transport in metal AM using mass and momentum balance equations [28]. Roberts et al. developed a 3D FEM to predict the temperature field considering the multilayer aspect of metal AM. They employed the element birth and death method to capture the addition of layers [29]. Antony et al. developed a FEM to predict the temperature distribution in AISI 316L. They have studied the effect of scan speed, laser power and beam size on temperature distribution [30]. Zohdi et al. developed a discrete element model to simulate the deposition and heat transfer involved in the process of SLM [31].

Mirkoohi et al. proposed a 2D physics-based analytical model to predict the temperature field in the metal AM process. In the modeling of the temperature field, the effects of melting-solidification phase change, layer addition, and temperature-sensitive material properties are considered. The FEM and also experimental work are used to validate the proposed analytical model [32]. Pinkerton et al. proposed an analytical model to model the geometry of the melt pool. The model considers the pool boundaries orthogonal to the direction of motion as arcs of a circle to account for maximum surface tension and elongation of the melt pool at high speeds [33]. Peyer et al. employed both analytical and numerical approaches to predict the shapes of the manufactured structure and thermal loadings in the direct metal deposition process. In their modeling, the powder temperature is predicted using an analytical model, and an analytical-numerical model is used to predict the geometry [34]. Yang et al. proposed a semi-analytical model to predict the temperature during the SLM process. A point heat source model is discretized to laser scanning vectors and the evolution of the temperature is predicted using superposition of the temperature field due to the discretized scanning vectors [3]. Carslaw and Jaeger employed separation of variables approach to predict the temperature field for the moving point heat source in a semi-infinite body [35]. 

The complex three-dimensional parts can be manufactured using the SLM process by locally melting the desired portion of the powders, layer by layer [36]. The necessary heat for melting the powders is provided by a laser. The laser–matter interaction is a crucial part in the modeling of the SLM process. Various heat source modeling approaches exist in the literature to simulate the temperature field during the SLM process. Each of models has its own limitations, such as ignoring the temperature sensitivity thermal material properties, ignoring the multi-layer aspect of metal AM, ignoring the solid-state phase change. In this paper, five different analytical heat source modeling approaches are introduced. The laser power and the scan speed are divided into three regions of high, medium and low to investigate the ability of each of models in prediction of temperature field. The predicted temperature fields are validated by experimental measurement of melt pool geometry [37,38,39]. 

Moreover, the effect of process parameters on the balling effect is also discussed. In this paper, the single-track temperature field is predicted by considering the laser power absorptivity since it has a direct influence on predicted melt pool temperature and geometry. Moreover, in the presence of the laser, the temperature of the build part goes up to thousands of degrees and also the material undergoes repeated heating and cooling, so, the material properties could change drastically. Consequently, it is important to consider the temperature-dependent thermal material properties. Furthermore, the multi-layer aspect of metal AM could change the heat transfer mechanisms. As a result, it has a substantial influence on the predicted temperature field. Accordingly, the effect of the addition of layers is also considered in the modeling as explained in previous work [32]. Due to the repeated melting and solidification process during the metal AM process, the solid-state phase change is considered by modifying the heat capacity using the latent heat of melting. It should be noted that the proposed models can be used to predict the temperature in laser-based metal additive manufacturing configurations of either direct metal deposition or selective laser melting.

## 2. Methodology

**Figure 1:**
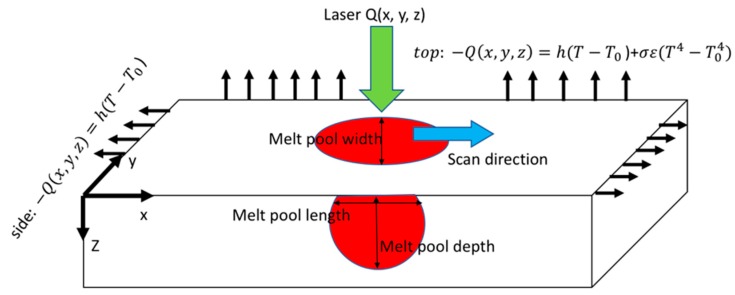
Illustration of the heat transfer model in laser bed metal additive manufacturing process.

In this section, five different heat source modeling approaches are introduced to predict the temperature field in laser-based metal AM process of SLM. The temperature field is predicted using both steady-state moving heat source and transient moving heat source in a semi-infinite body. As shown in Figure 1, the laser moves along the scan direction (x-axis) and deposited its energy to melt the metallic powders. The heat loss due to convection and radiation is not considered in this modeling.

Heat conduction in a homogeneous solid is governed by the linear partial differential equation:

(1) $$\frac{\partial u\rho }{\partial t}+\frac{\partial \rho hV}{\partial x}=\nabla .\left(k\nabla T\right)+\dot{q}$$

where u represents the internal energy, h is the enthalpy, ρ is the density, k is the conductivity, $\dot{q}$ is a volumetric heat source, *T* is the temperature and V is the speed of either the heat source or the medium. 

The x direction corresponds to the constant speed of a moving heat source. Also, y is directed inside the processed material, and z, the direction perpendicular to x in the plane of the processed material surface. The first term in Equation (1) on the left-hand side represents the change of internal energy and the second is a convective term. On the right-hand side, there is the conductive term and a heat source or sink. For V=0, this equation becomes the heat conduction equation, given that d*u* = *C*d*T*, with *C* being the heat capacity:

(2) $$C\frac{\partial \rho T}{\partial t}+\frac{\partial \rho hV}{\partial x}=\nabla .\left(k\nabla T\right)+\dot{q}$$

The steady state equation with constant velocity V, can be simplified using the continuity equation:

(3) $$C\frac{\partial \rho }{\partial t}+\frac{\partial \rho V}{\partial x}=0$$

Resulting in:

(4) $$\rho C\left(T\right)V\frac{\partial T}{\partial x}+\frac{\partial \rho hV}{\partial x}=\nabla .\left(k\left(T\right)\nabla T\right)+\dot{q}$$

given that d*u* = d*h* = *C*d*T*.

The convection-diffusion equation becomes the differential equation of heat conduction which can be expressed as:

(5) $$\frac{\partial^{2}T}{\partial x^{2}}+\frac{\partial^{2}T}{\partial y^{2}}+\frac{\partial^{2}T}{\partial z^{2}}+\frac{1}{k}Q\left(x,y,z,t\right)=\frac{1}{D}\frac{\partial T}{\partial t}$$

where *T* ≡T(x,y,z,t). k is thermal conductivity, and D is the thermal diffusivity. The heat source Q is related to the equivalent volumetric source Q(x,y,z,t) ($W/m^{3})$ by the delta function notation as:

(6) $$Q\left(x,y,z,t\right)=\;Q\;\delta \left(x-Vt\right)\delta \left(y-y_{0}\right)\delta \left(z-z_{0}\right)$$

where δ denotes the Dirac delta function. 

In order to consider the moving heat source, it is assumed that the coordinate system transfers from the x, y,z fixed coordinate system to ζ, y,z coordinate moving by using the transformation:

(7) ζ=x−Vt

Using the abovementioned transformation, the heat conduction equation for the moving coordinate system can be written as:

(8) $$\frac{\partial^{2}T}{\partial \zeta^{2}}+\frac{\partial^{2}T}{\partial y^{2}}+\frac{\partial^{2}T}{\partial z^{2}}+\frac{1}{k}Q\delta \left(\zeta \right)\delta \left(y\right)\delta \left(z\right)=\frac{1}{D}\left(\frac{\partial T}{\partial t}-V\frac{\partial T}{\partial \zeta }\right)$$

### 2.1. Steady State Moving Point Heat Source

Equation (8) can be solved by the assumption of the quasi-stationary condition by setting $\frac{\partial T}{\partial t}=0$ [35]. Using the separation of variables, the closed form solution of the temperature field can be obtained as:

(9) $$T=\;\frac{P\eta }{4\pi kR}\exp\frac{-V\left(R-x\right)}{2D}+T_{0}$$

where P is the laser power, η represents the absorption coefficient, *k* is thermal conductivity and assumed to be temperature dependent, V is scan speed, $T_{0}\;$is the initial temperature.

*R* is the radial distance from the heat source which can be calculated as:

(10) $$R\;=\;\sqrt{x^{2}+y^{2}+z^{2}}$$

D is thermal diffusivity and can be obtained from:

(11) $$D\left(T\right)=\frac{k\left(T\right)}{\rho C_{p}^{m}\left(T\right)}$$

where ρ is material density and $C_{p}^{m}\left(T\right)$ is the modified heat capacity. The melting/solidification phase transformation take place during the AM process and it has a profound effect on melt pool geometry. This is considered using modified heat capacity.

(12) $$C_{p}^{m}=\;C_{p}\left(T\right)+L_{f}\frac{\partial f}{\partial T}$$

In which $C_{p}\left(T\right)$ is temperature dependent specific heat, $L_{f}$ is latent heat of fusion, and f is liquid fraction which can be calculated from:

(13) $$f=\{\begin{array}{c} 0,\;\;T<T_{s}\\ \frac{T-T_{s}}{T_{L}-T_{s}},\;\;T_{s}<T<T_{L}\\ 1,\;\;\;\;\;\;\;\;\;\;\;T>T_{L} \end{array}$$

where, $T_{s}\;$is solidus temperature and $T_{L}$ is liquidus temperature.

### 2.2. Transient Moving Point Heat Source

The transient point heat source solution is developed by Carslaw and Jaeger [35] using the differential equation of heat conduction (Equation (8)) as the following:

(14) $$T=\;\frac{P\eta }{8\rho C_{p}^{m}{\left(\pi Dt\right)}^{3/2}}\int_{0}^{t}\frac{exp\left(-\frac{{\left(\left(x-x^{\prime }\right)-V\left(t-t^{\prime }\right)\right)}^{2}+{\left(y-y^{\prime }\right)}^{2}+{\left(z-z^{\prime }\right)}^{2}}{4D\left(t-t^{\prime }\right)}\right)}{{\left(t-t^{\prime }\right)}^{3/2}}dt^{\prime }+T_{0}$$

Equation (14) gives the temperature field at position (*x*,*y*,*z*) at time t due to an instantaneous unit heat source applied at position ($x^{\prime },y^{\prime },z^{\text{’}})\;$ at time $t^{\prime }$.

Where P is the laser power, η represents the absorption coefficient, ρ is density, $C_{p}^{m}$ is the modified heat capacity to account for solid state phase change as explained in Equations (12) and (13). V is scan speed, D is thermal diffusivity, and $T_{0}\;$is the initial temperature.

### 2.3. Transient Semi-Elliptical Moving Heat Source

A three-dimensional (3D) semi-ellipsoidal heat transfer model is used to predict the temperature field and melt pool geometry in metal AM processes. The proposed model can be used to predict the temperature in laser-based metal additive manufacturing configurations of either direct metal deposition or selective laser melting. 

**Figure 2:**
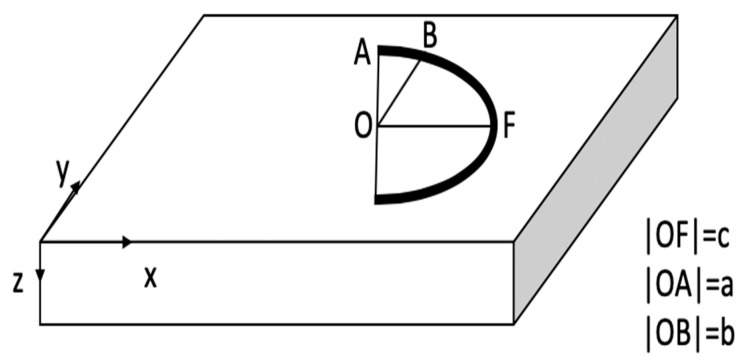
Heat source geometry.

The 3D ellipsoidal heat source model is introduced by Goldak et al. [40] where the heat flux can be calculated as:

(15) $$q\left(x,y,z\right)=\frac{6\sqrt{3}\;\eta P}{abc\pi \sqrt{\pi }}exp(-\frac{3x^{2}}{c^{2}}-\frac{3y^{2}}{a^{2}}-\frac{3z^{2}}{b^{2}})$$

where *P* is the laser power, η is laser absorptivity, *a*, *b*, and *c* are the heat source geometry parameters, as shown in Figure 2. 

The solution of temperature for ellipsoidal moving heat source from *t*’ = 0 to t for a semi-infinite body in a dimensionless form is given as [41]*:*

(16) $$\frac{\theta }{n}=\frac{1}{\sqrt{2\pi }}\int_{0}^{\frac{v^{2}t}{2k}}\frac{d\tau }{\sqrt{\tau +u_{a}^{2}}\sqrt{\tau +u_{b}^{2}}}\left(\frac{A_{1}}{\sqrt{\tau +u_{c}^{2}}}\right)$$

where,

(17) $$A_{1}=exp\left(-\frac{{\left(\xi +\tau \right)}^{2}}{2\left(\tau +u_{c}^{2}\right)}-\frac{\psi^{2}}{2\left(\tau +u_{a}^{2}\right)}-\frac{\lambda^{2}}{2\left(\tau +u_{b}^{2}\right)}\right)$$

The dimensionless parameters are defined as:

(18) $$\xi =\frac{Vx}{2D},\;\psi =\frac{Vy}{2D}\;,\;\;\lambda =\frac{Vz}{2D}$$

where V is scan speed, and D is thermal diffusivity which can be obtained from Equation (11).

(19) $$\tau =\frac{V^{2}\left(t-t^{\prime }\right)}{2D}$$

(20) $$u_{a}=Va2\sqrt{6}\;D,\;u_{b}=Vb2\sqrt{6}\;D,\;u_{c}=Vc2\sqrt{6}\;D$$

(21) $$n=\frac{P\eta V}{4\pi D^{2}\rho C\left(T_{m}-T_{0}\right)}$$

(22) $$T=\;\frac{P\eta V}{4\pi D^{2}\rho C}\times \frac{1}{\sqrt{2\pi }}\int_{0}^{\frac{V^{2}t}{2k}}\frac{d\tau }{\sqrt{\tau +u_{a}^{2}}\sqrt{\tau +u_{b}^{2}}}\left(\frac{A_{1}}{\sqrt{\tau +u_{c}^{2}}}\right)+T_{0}$$

### 2.4. Transient Double Elliptical Moving Heat Source

**Figure 3:**
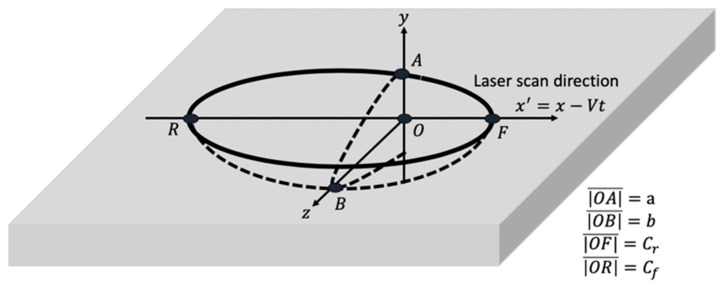
Illustration of the double elliptical heat source model.

The 3D double ellipsoidal heat source model is developed by Goldak et al. [40] as follows:

(23) $$q\left(x,y,z,t\right)=\frac{6P\eta \sqrt{3}}{\pi \sqrt{\pi }ab}\left\{\begin{array}{c} \frac{f_{f}}{c_{f}}exp-3\frac{{\left(x-Vt\right)}^{2}}{c_{f}{}^{2}}-3\frac{y^{2}}{a^{2}}-3\frac{z^{2}}{b^{2}}\;for\;x>vt\\ \frac{f_{r}}{c_{r}}exp-3\frac{{\left(x-Vt\right)}^{2}}{c_{r}{}^{2}}-3\frac{y^{2}}{a^{2}}-3\frac{z^{2}}{b^{2}}\;for\;x<vt \end{array}\right\}$$

where P is laser power energy, η  is laser absorptivity, $a,\;b,\;c_{r}\;\mathrm{and}\;c_{f}$ are the respective radii of the sides, rear and front of the ellipsoid as shown in Figure 3. V is the scan speed.

In the previous section, five different 3D heat source models known as steady state moving point heat source, transient moving point heat source, transient semi-elliptical moving heat source, transient double elliptical moving heat source, and uniform moving point heat source are introduced. The accuracy and applicability of these models are investigated for the different range of process parameters such as scan speed and laser power. The predicted temperature field from each model is validated with experimental results of the melt pool geometry.

$f_{r}$ and $f_{f}$ are the portion of the heat deposited, respectively, in the front and rear ellipsoid (with $f_{r}$ + $f_{f}$ = 2). In order to fit the results to experimental data, calibration of the double-ellipsoidal heat source model requires adjustment of six parameters including $\eta ,a,b,c_{r},$ $c_{f},\;\mathrm{and}\;f_{r}.$ Some researchers tried to reduce this number by applying a constraint to the model as [41,42,43]:

(24) $$\frac{f_{f}}{c_{f}}=\frac{f_{r}}{c_{r}}$$

This constraint guarantees the continuity of the function q across the x=Vt plane at any time, t, to yield:

(25) $$f_{f}=\frac{\alpha c_{f}}{c_{f}+c_{r}}$$

α=3 correlates to the choice of $f_{f}=0.6$ as suggested by Goldak et al. [40], and should be used as a default value when enough experimental data are not available. It should be noted that the temperature field induced by double double-ellipsoidal heat source, q,  is always continuous. In other words, it is independent of the value of α.

The Green’s function approach is used to solve the differential equation of heat conduction (Equation (8)) to derive the temperature field for the case of double elliptical heat source as:

(26) $$T\left(x,y,z,t\right)=T_{0}+\frac{3P\eta \sqrt{3}}{\pi \sqrt{\pi }\;\rho C}\times \int_{0}^{t}\frac{\exp\left[-3\frac{y^{2}}{12D\left(t-t^{\prime }\right)+a^{2}}-3\frac{z^{2}}{12D\left(t-t^{\prime }\right)+b^{2}}\right]}{\sqrt{12D\left(t-t^{\prime }\right)+a^{2}}\times \sqrt{12D\left(t-t^{\prime }\right)+b^{2}}}\times \left[f_{r}A_{r}\left(1-B_{r}\right)+f_{f}A_{f}\left(1+B_{f}\right)\right]dt^{\prime }$$

where,

(27) $$A_{i}=A\left(x,t,t^{\prime };c_{i}\right)=\frac{\exp\left[-3\frac{{\left(x-Vt\right)}^{2}}{12D\left(t-t^{\prime }\right)+c_{i}{}^{2}}\right]}{\sqrt{12D\left(t-t^{\prime }\right)+c_{i}{}^{2}}}$$

(28) $$B_{i}=B\left(x,t,t^{\prime };c_{i}\right)=erf\frac{c_{i}\left(x-Vt\right)}{2\sqrt{12D\left(t-t^{\prime }\right)+c_{i}{}^{2}}\sqrt{D\left(t-t^{\prime }\right)}}$$

The equation gives the temperature field at position (*x*,*y*,*z*) at time t due to an instantaneous unit heat source applied at position ($x^{\prime },y^{\prime },z^{\text{’}})\;\mathrm{at}\;\mathrm{time}\;t^{\prime }$. The index *i* denotes front, *f*, or rear, *r*.

### 2.5. Transient Uniform Moving Heat Source

The heat flux q(x,y,z) at any point (x,y,z) for a uniform heat source is given as [35]:

(29) $$q\left(x,y,z\right)=\frac{P\eta }{4\;abc}\left\{\begin{array}{c} -a<x<a\\ -b<y<b\\ 0<z<c \end{array}\right\}$$

where *P* is the laser power, η  is laser absorptivity, *a*, *b*, and *c* are the heat source geometry parameters.

As for the other types of the heat sources, the solution of the temperature field is based on an instantaneous point source in a fixed coordinate:

(30) $$T=T_{0}-\frac{P\eta }{2^{5}\rho C\;abc}\int_{0}^{t}Erfh(x+V(t-t^{\prime }),a,t^{\prime })\times Erfh\left(y,b,t^{\prime }\right)\times Erfh\left(z,c,t^{\prime }\right)dt^{\prime }$$

with ${\mathrm{Fo}}_{s}$ the Fourier number based on s and $t-t^{\text{′}}$ as length and time, respectively,

(31) $$Erfh\left(x,s,t^{\prime }\right)≜Erf\left(\frac{x-s}{\sqrt{4D\left(t-t^{\prime }\right)}}\right)-Erf\left(\frac{x+s}{\sqrt{4D\left(t-t^{\prime }\right)}}\right)=Erf(\frac{\sqrt{Fo_{s}}}{2}\left(\frac{x}{s}-1\right)-Erf\left(\frac{\sqrt{Fo_{s}}}{2}\left(\frac{x}{s}+1\right)\right)≜Erf\left(\frac{x}{s},\;Fo_{s}\right)$$

## 3. Results and Discussion

In the previous section, five different 3D heat source models known as steady state moving point heat source, transient moving point heat source, transient semi-elliptical moving heat source, transient double elliptical moving heat source, and uniform moving point heat source are introduced. The accuracy and applicability of these models are investigated for the different range of process parameters such as scan speed and laser power. The predicted temperature field from each model is validated with experimental results of the melt pool geometry.

### 3.1. Experimental Procedure

Eight different process parameters are selected from three different works in the literature to validate the introduced models. The material is Ti-6Al-4V. The laser power varies from 20−500 W, the scan speed varies from 6−1200 mm/s. The process parameters are listed in Table 1.

The first four data are selected from the work of Fu et al. [37]. In this work, a continuous laser of type Nd:YAG with the wavelength of 1.06 µm is used to melt the Ti-6Al-4V metallic powders with a layer thickness of 30 µm during the single track SLM process. The layer thickness is the deposited height of metallic powders in each layer. The wavelength of the laser and the material determine the absorption coefficient. Based on the reported wavelength and the material which is Ti-6Al-4V, the absorption coefficient would be 0.77. The laser power varies from 20 W to 80 W and the scan speed is fixed at 200 mm/s. The laser spot radius is 30 µm. The melt pool geometry is measured using optical microscopy based on the solidified microstructure.

The fifth experimental data in Table 1 is obtained from the work of Yiqun et al. [38]. In this work, a Ti-6Al-4V sample is built using laser melting deposition process. The laser power is 500 W and scan speed is 6 mm/s. Moreover, the layer thickness and laser spot radius are 45 µm and 26 µm, respectively. The melt pool is capture using thermal imager. More information about experimental data can be obtained in [38].

The last three experimental data are obtained from the work of Soylemez [39]. A continuous laser with a wavelength of 1.06 µm is used to build the Ti-6Al-4V parts with the fixed laser power of 300 W. The scan speed varies from 400 mm/s to 1200 mm/s. Furthermore, the layer thickness and laser spot radius are 30 µm and 50 µm, respectively. The samples are prepared using a polishing and etching process to measure the melt pool geometry under an optical microscope, as reported in [39].

The wide range of process parameters are selected to validated each of the analytical models.

### 3.2. Modeling Results

The temperature field is predicted using five different heat source models. The predicted melt pool geometry such as melt pool depth width and length is compared to experimental measurement. Different combinations of laser power and scan speed are used to cover all the ranges of process parameters from low to high to investigate the applicability of each model at different process parameter ranges. Due to the high temperature gradient in the SLM process, the thermal material properties such as thermal conductivity and specific heat vary significantly. Therefore, the thermal material properties of the Ti-6Al-4V are considered to be temperature-dependent as listed in Table 2. Also, during the SLM process, the part undergoes cyclic melting and solidification process, and this is considered in the modeling by modifying the heat capacity using latent heat of melting. Moreover, the deposition of the metallic powders layer by layer could change the thermodynamic and heat transfer mechanisms. Consequently, it is important to consider the layering addition in the modeling of the temperature field. 

#### 3.2.1. Comparison of Different Heat Source Geometry

**Figure 4:**
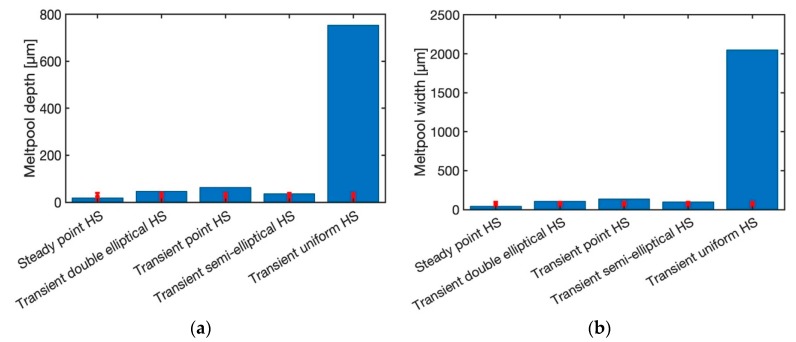
Predicted melt pool geometry with the laser power of 20 W and scan speed of 200 mm/s (**a**) melt pool depth, (**b**) melt pool width (Sample 1 in Table 1).

**Figure 5:**
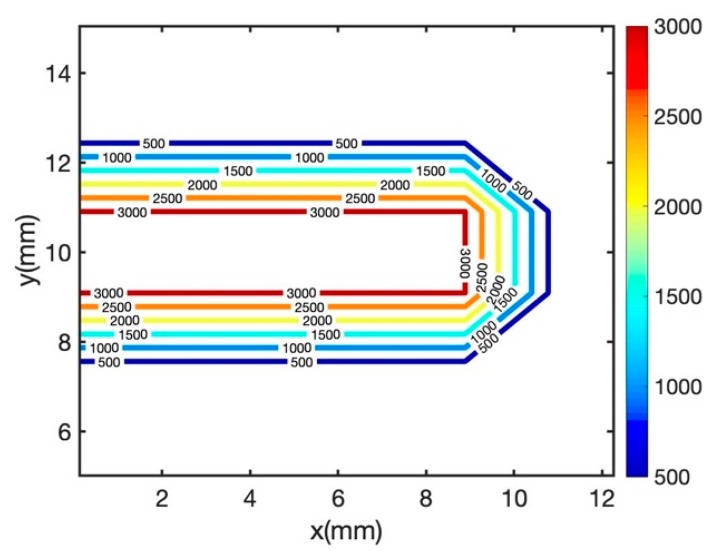
Illustration of the melt pool geometry using uniform moving heat source.

**Figure 6:**
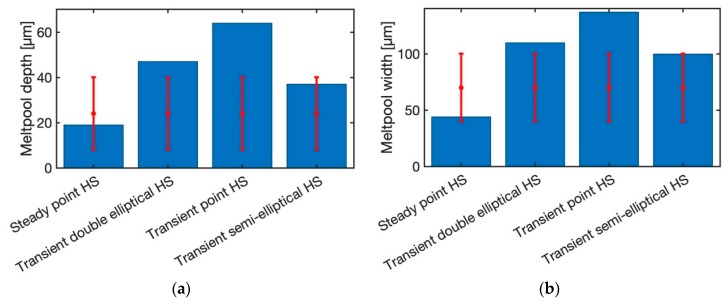
Predicted melt pool geometry with the laser power of 20 W and scan speed of 200 mm/s (**a**) melt pool depth, (**b**) melt pool width (Sample 1 in Table 1).

Figure 4 illustrates the predicted melt pool depth and width for the laser power of 20 W and the scan speed of 200 mm/s. The laser spot radius is 26 µm and the layer thickness is 30 µm, the same as the experimental procedure. Moreover, the absorption ratio is 0.77. The predicted melt pool depth and width with uniform moving heat source are overestimated when compared to experimental measurements. The main reason is that the geometry of the uniform heat source is more like a rectangular shape as shown in Figure 5. However, the actual melt pool geometry from the experiment has a circular and elliptical shape in most of the cases. Figure 6 is the same plot as Figure 4, but the transient uniform heat source is omitted from the plot to better illustrate the comparison of the predicted and measured melt pool depth and width for steady state point heat source (HS), transient double elliptical HS, transient point HS, and transient semi-elliptical HS.

The predicted melt pool depth is accurately predicted with a steady state point HS model and transient semi-elliptical heat source. A transient double elliptical heat source has predicted the melt pool depth with 12.5% error. Moreover, the transient point heat source model has predicted the melt pool depth with 50% error. The reason for a high amount of error in transient heat source model is that the predicted melt pool geometry is varying with time. Since this is a transient model, due to the passing of time more heat would be conducted through the solid which would result in lower melt pool geometry prediction. It should be noted that the point heat source approach is usually used for surface laser treatment processes such as laser hardening and laser conductive melting. Also, it is good to represent the absorption of laser radiation in the metal surface. Consequently, at different time steps the melt pool geometry would vary slightly. In this paper, in order to be consistent in the modeling, the predicted melt pool geometry is obtained immediately after the radiation of the laser.

The melt pool width is also predicted for all five heat source models. The uniform heat source model is predicted for all the selected process parameters in Table 1. As explained before, the transient uniform HS could not predict the melt pool geometry with a reasonable range of error. Since the predicted melt pool geometry using this heat source geometry is much higher than the experimental measurements, the results are not shown in this paper.

The melt pool width is captured by steady-state moving HS, transient double elliptical HS, transient point HS, and transient semi-elliptical HS with the maximum error of 0%, 10%, 37%, 0%, respectively. 

Predicted melt pool geometry using steady state point HS, transient double elliptical HS, transient point HS, and transient semi-elliptical HS for all the eight samples are listed in Table 3.

#### 3.2.2. Region of Applicability of Each Model Based on Laser Power and Scan Speed

**Figure 7:**
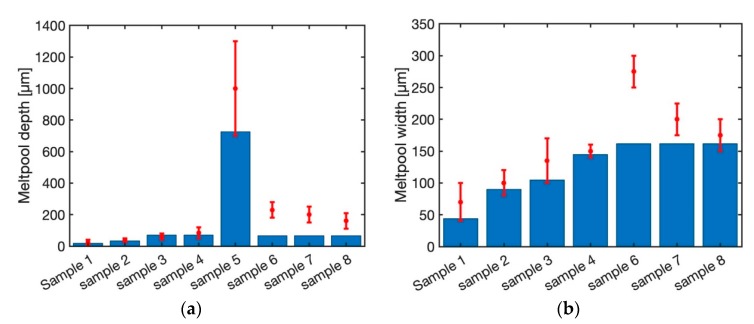
Predicted melt pool (**a**) depth and (**b**) width using steady state moving HS.

Figure 7 illustrates the predicted melt pool depth and width using steady-state moving point heat source approach for different laser power and scan speed as listed in Table 1. The material for all the samples is Ti-6Al-4V. Sample 1 through sample 4 is made using the laser power of 20 W, 40 W, 60 W and 80 W with the fixed scan speed of 200 mm/s. As shown in this figure, the predicted melt pool depth and width using a steady state moving point heat source approach are within the range of experimental measurements. Sample 5 is built using laser power of 500 W and the scan speed of 6 mm/s. The predicted melt pool depth is within the range of experimental measurement. The melt pool width for this sample is not reported in the literature. Sample 6 through sample 8 are built using a fixed laser power of 300 W with the scan speed of 400 mm/s, 800 mm/s, and 1200 mm/s, respectively. The predicted melt pool depth and width for these three samples are equal. The predicted melt pool depth is 67 µm and the predicted melt pool width is 162 µm. This shows that the steady state moving point heat source approach does not have the ability to predict the temperature field for high laser speeds. This is because of the behavior of the exponential term. As the power value (laser speed) of the exponential term increase, the output of the function will be less sensitive to the power value. Consequently, in these cases where the magnitude of the scan speed is high, the temperature field does not change.

**Figure 8:**
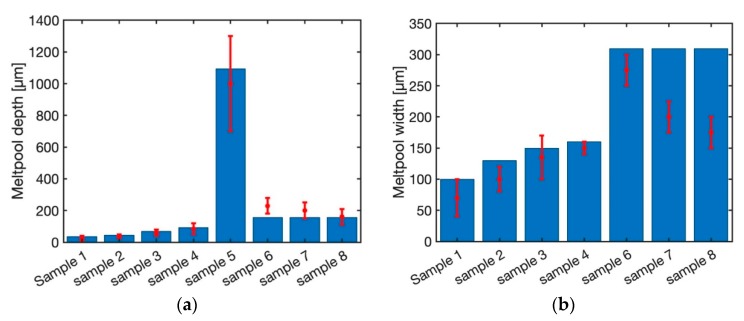
Predicted melt pool (**a**) depth and (**b**) width using semi-elliptical moving HS.

Figure 8 demonstrates the predicted melt pool geometry using the semi-elliptical heat source model. The predicted melt pool widths for the first four samples are within the range of experimental measurements. The melt pool width and depth do not change for the samples 6 through 8. The main reason could be the effect of heat source geometry. As explained by Goldak et al. [40] the heat source parameters should be calibrated using experimental data. Since the goal of this work is the comparison of the heat source models, the authors tried to be consistent in the modeling and comparison. So, the same calibration based on experimental data is used for all the samples and models.

**Figure 9:**
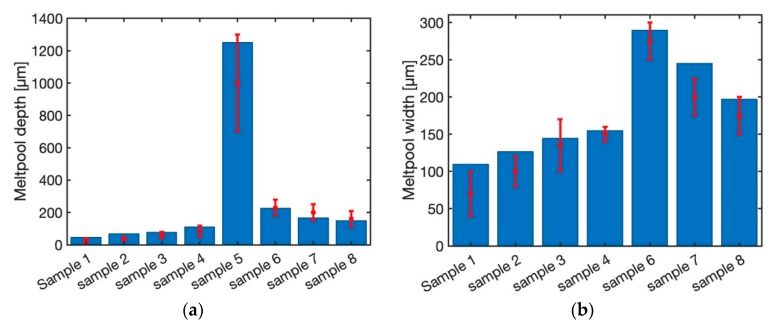
Predicted melt pool (**a**) depth and (**b**) width using double elliptical moving HS.

Figure 9 demonstrates the predicted melt pool depth and width for 8 samples using the double elliptical moving heat source approach. The predicted melt pool depth and width for all the samples are within the range of experimental measurements. This shows that the double elliptical moving heat source could be used for all the range of laser powers and scan speeds. The main reason is that the actual melt pool geometry from the experimentation resembles the ellipsoidal shape. Consequently, the double elliptical moving heat source could simulate the melt pool geometry quite well. 

**Figure 10:**
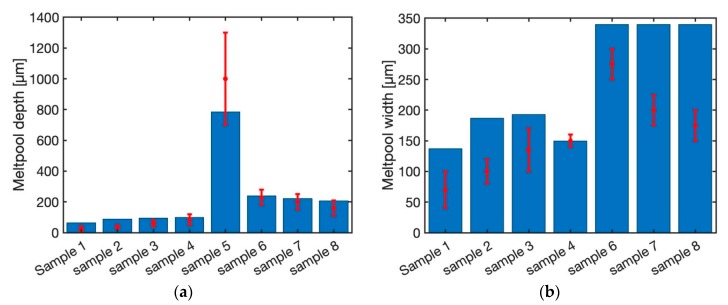
Predicted melt pool (**a**) depth and (**b**) width using transient moving point HS.

Figure 10 depicts the melt pool depth and width using the transient moving point heat source approach. The melt pool depth is well captured using this approach for all the samples. Moreover, predicted melt pool width for the first four samples (sample 1 through 4) are predicted with the maximum error of 15%. The predicted melt pool width for sample 6 through 8 does not change. This is due to the existence of the exponential term in the modeling of the temperature field using the transient moving heat source approach, as explained before. As a result, the transient moving point heat source approach could not be used for the prediction of the temperature field at high speeds.

#### 3.2.3. Effect of Process Parameters on Balling Effect

During the SLM process, the molten powder possesses a shrinking tendency to decrease the surface energy induced by surface tension [44,45]. Thus, the balling phenomenon is very easily formed during the SLM process, which is detrimental to the quality of the SLM-processed part and impede the further development of SLM technology. The balling effect hinders the quality and performance of the final part due to the increase of the surface roughness. This would result in post-processing procedure to polish the samples. These procedures are not only time consuming, but also costly. The other disadvantageous of the balling effect during the AM process is the formation of the pores between the balls which result in poor mechanical properties [46]. The balling effect could happen for two main reasons. One is the formation of the balls due to the inadequate laser energy input with little liquid content. And the other due to molten pool splashes induced by high scan speed. Consequently, the laser power and scan speed have a direct influence on balling effect. The balling effect can be determined from the molten pool geometry. A large molten pool length to depth ratio ($\frac{L}{D}>\pi )$ would result in the formation of the balls [47].

**Figure 11:**
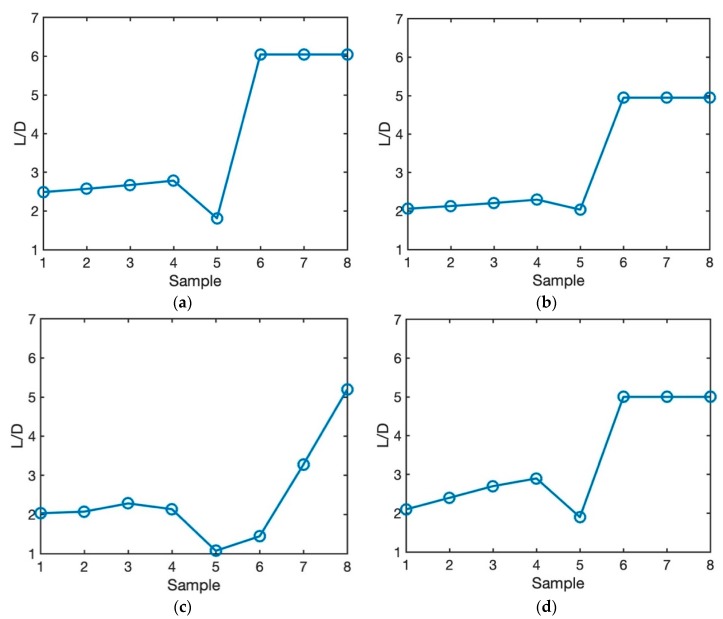
Prediction of balling for 8 different samples as listed in Table 1 using (**a**) steady state moving point heat source, (**b**) semi-elliptical moving heat source, (**c**) double elliptical moving heat source, and (**d**) transient moving point heat source.

In this section, the effect of process parameters such as laser power and scan speed on balling effect is investigated for eight different process parameters as listed in Table 1. As shown in Figure 11a, the balling effect is predicted using steady state moving point heat source approach. $\frac{L}{D}<\pi$ is predicted for samples 1 through 5, which indicates that the process parameters are selected appropriately resulting in the formation of no balls. This is also indicated in the experimental measurements. However, sample 6 through 8 experience the balling effect since the predicted $\frac{L}{D}\;\mathrm{is}\;\mathrm{greater}\;\mathrm{then}\;\pi$. This is also observed in the experimentation as reported by Soylemez [39].

Figure 11b illustrates the predicted balling effect using a semi-elliptical heat source model. This model also correctly predicts that the first five samples do not experience the balling effect. Moreover, this model predicts the formation of the balls for samples 6 through 8 due to the large molten pool length to depth ratio.

Figure 11c,d depict the predicted balling effect employing a double elliptical heat source model, and transient moving point heat source model, respectively. Both of these models accurately predict no formation of the balls for the first five samples, and formation of the balls for samples 6 through 8.

As shown in these figures, the combination of process parameters such as scan speed and laser power have a substantial effect on the formation of the balls. The process parameters can be optimized to prevent any formation of the balls during the AM process.

## 4. Conclusions

The laser–matter interaction is a crucial physical phenomenon in the SLM process. In this paper, five different heat source models known as steady state moving point heat source, semi-elliptical moving point heat source, double elliptical moving point heat source, transient moving point heat source, and uniform moving heat source are introduced in order to predict the in-process temperature field in the SLM process for Ti-6Al-4V samples. Due to the large temperature gradient during the SLM process, the magnitude of the thermal material properties varies significantly. As a result, the material properties are considered to be temperature dependent. Moreover, the build part undergoes cyclic melting and solidification process which impacts the temperature field. Consequently, the solid-state phase change is considered by modifying the heat capacity using the latent heat of melting. During the SLM process, the part is built layer by layer. This would have a substantial impact on heat transfer mechanisms. Accordingly, the effect of layering addition is also considered by considering the temperature history and interaction of the layers in the modeling of the temperature field using five different approaches. Predicted melt pool geometries from these models are compared to experimental work. Wide range of process parameters is selected to determine the capability of each of models. The laser power varies from 20 W to 500 W, and the scan speed varies from 6 mm/s to 1200 mm/s. 

The predicted melt pool geometry using uniform moving heat source is significantly above the experimental measurements. This is due to the fact that the uniform heat source resembles a rectangle shape. However, the actual molten pool geometry from the experiments is more like an elliptical shape or a circular shape. 

The steady-state moving point heat source, semi-elliptical heat source, and transient moving point heat source could predict the melt pool geometry quite well for low and medium scan speeds. However, at high speeds, no changes in melt pool geometry are observed. This is due to the behavior of the exponential term in the modeling of these heat sources. 

The double elliptical model could predict the melt pool geometry for all the ranges of the process parameters. This is due to the fact that the double elliptical heat source resembles the actual melt pool geometry from experimentation. As a result, it can predict the temperature field quite well. 

Furthermore, the effect of process parameters on the balling effect is investigated. Steady-state moving point heat source, semi-elliptical moving point heat source, double elliptical moving point heat source, and transient moving point heat source models are used to predict the formation of the balls in the SLM process. All the models could predict the formation of the balls for 8 different samples. The results are also compared to the experimental work explained in the literature. It is shown that the combination of scan speed and laser power is the main reason for the existence of the balls. Consequently, these process parameters should be selected in a way to prevent any formation of the balls.

## Figures and Tables

**Table 1:** List of process parameters used for model validation.

Sample	1 [37]	2 [37]	3 [37]	4 [37]	5 [38]	6 [39]	7 [39]	8 [39]
Laser power (W)	20	40	60	80	500	300	300	300
Scan speed (mm/s)	200	200	200	200	6	400	800	1200
Laser spot radius (µm)	26	26	26	26	26	50	50	50
Layer thickness (µm)	30	30	30	30	45	30	30	30
Absorption ratio	0.77	0.77	0.77	0.77	0.77	0.77	0.77	0.77

**Table 2:** List of temperature-dependent thermal material properties of Ti-6Al-4V. (Temperature in °C).

Properties	Ti-6Al-4V
Liquidus temperature (°C)	1655
Solidus temperature (°C)	1605
Thermal conductivity (W/m°C)	$\{\begin{array}{l} K_{s}=1.57+1.6\times 10^{-2}T-1\times 10^{-6}T^{2}\;995\;<\;T\;<\;1655\\ K_{l}=\;33.4\;T\;=\;1655\\ K_{l}=\;34.6\;T\;=\;1655 \end{array}$
Specific Heat (J/kg°C)	$\{\begin{array}{l} C_{p}=492.4+0.025T-4.18\times 10^{-6}T^{2}\;995\;<\;T\;<\;1655\\ C_{p}=830\;T\;>\;1655 \end{array}$
Density (kg/m^3^)	$\{\begin{array}{l} \rho_{s}=4.42-15.4\times 10^{-2}T\;995\;<\;T\;<\;1655\\ \rho_{l}=3.92-68.0\times 10^{-2}\left(T-1020\right)\;T\;>\;1655 \end{array}$
Latent heat (J/g)	286

**Table 3:** Predicted melt pool geometry for eight different samples.

Sample	Steady State Moving Point Heat Source (HS)		Transient Moving Point HS		Semi-Elliptical HS		Double-Elliptical HS		Experiment	
	Depth (µm)	Width (µm)	Depth (µm)	Width (µm)	Depth (µm)	Width (µm)	Depth (µm)	Width (µm)	Depth (µm)	Width (µm)
1	19	44	64	137	37	100	47	110	24−40 [37]	70−100 [37]
2	33	90	89	187	46	130	68	127	37−50 [37]	80−120 [37]
3	71	105	95	193	68	150	78	145	50−80 [37]	110−170 [37]
4	72	145	100	150	94	160	111	155	85−110 [37]	140−160 [37]
5	727	--	786	--	1095	--	1252	--	700−1300 [38]	--
6	67	162	241	340	156	310	227	290	190−280 [39]	250−300 [39]
7	67	162	222	340	156	310	168	245	150−250 [39]	185−225 [39]
8	67	162	206	340	156	310	150	197	110−210 [39]	150−200 [39]
